# The porcine piRNA transcriptome response to Senecavirus a infection

**DOI:** 10.3389/fvets.2023.1126277

**Published:** 2023-05-30

**Authors:** Chen Wang, Yanxi Chen, Xiwang Yang, Yunsha Du, Zhiwen Xu, Yuancheng Zhou, Xu Yang, Xuetao Wang, Chuanming Zhang, Shuwei Li, Yijun Yang, Wenting Li, Xiao Liu

**Affiliations:** ^1^Southwest University, College of Veterinary Medicine, Chongqing, China; ^2^Animal Biotechnology Center, College of Veterinary Medicine, Sichuan Agricultural University, Chengdu, China; ^3^Veterinary Biologicals Engineering and Technology Research Center of Sichuan Province, Animtech Bioengineering CO., LTD., Chengdu, China; ^4^Livestock and Poultry Biological Products Key Laboratory of Sichuan Province, Sichuan Animal Science Academy, Chengdu, China; ^5^Animal Breeding and Genetics Key Laboratory of Sichuan Province, Sichuan Animal Science Academy, Chengdu, China; ^6^Department of Infectious and Tropical Diseases, The Second Affiliated Hospital of Hainan Medical University, Haikou, China; ^7^State Key Laboratory of Silkworm Genome Biology, Chongqing, China

**Keywords:** Senecavirus A (SVA), Piwi-interacting RNAs (piRNAs), pig kidney cells, virus infection, high-throughput sequencing

## Abstract

**Introduction:**

Senecavirus A (SVA) belongs to the genus *Senecavirus* in the family *Picornaviridae*. PIWI-interacting RNAs (piRNAs) are a class of small Ribonucleic Acids (RNAs) that have been found in mammalian cells in recent years. However, the expression profile of piRNAs in the host during SVA infection and their roles are poorly understood.

**Methods:**

Here, we found the significant differential expression of 173 piRNAs in SVA-infected porcine kidney (PK-15) cells using RNA-seq and 10 significant differentially expressed (DE) piRNAs were further verified by qRT-PCR.

**Results:**

GO annotation analysis showed that metabolism, proliferation, and differentiation were significantly activated after SVA infection. Kyoto Encyclopedia of Genes and Genomes (KEGG) analysis revealed that significant DE piRNAs were mainly enriched in AMPK pathway, Rap1 pathway, circadian rhythm and VEGF pathway. It was suggested that piRNAs may regulated antiviral immunity, intracellular homeostasis, and tumor activities during SVA infection. In addition, we found that the expression levels of the major piRNA-generating genes *BMAL1* and *CRY1* were significantly downregulated after SVA infection.

**Discussion:**

This suggests that SVA may affect circadian rhythm and promote apoptosis by inhibiting the major piRNA-generating genes *BMAL1* and *CRY1*. The piRNA transcriptome in PK-15 cells has never been reported before, and this study will further the understanding of the piRNA regulatory mechanisms underlying SVA infections.

## Highlights

- SVA infection altered the expression profile of piRNAs in PK-15 cells.
- 981 and 1,370 novel piRNAs were identified in SVA-infected and non-infected PK-15 cells.
- piRNAs may regulated antiviral immunity, intracellular homeostasis, and tumor activities during SVA infection.
- SVA infection may have an effect on circadian rhythm in PK-15 through inhibiting BMAL1 and CRY1 expression.

## Introduction

Senecavirus A (SVA) belongs to the *Picornaviridae* family and is the only member of the genus *Senecavirus* (1). In addition to *pigs*, SVA has also been found in *mice* and *buffaloes* (2, 3). In 2002, SVA was first identified in the United States as a cellular pollutant (4). In 2007, outbreaks of SVA were reported in Minnesota and Indiana, USA, and resulted in vesicular lesions in pigs (4, 5). At the end of 2014, SVA was reported to affect 80% of pig farms in Brazil (6, 7). In 2015, SVA began to spread rapidly around the world, marking a turning point in the spread of the epidemic (8). SVA has become widely distributed in the United States, Brazil, and Colombia (9). Since 2015 SVA has been widespread in Guangdong province of China (10), Thailand (11), Vietnam (12) and other Asian regions. The most typical clinical symptoms of SVA infected pigs are vesicular lesions in the skin and mucous membranes such as tongue and hoof crown. The clinical manifestations of SVA infection are very similar to FMDV, vesicular stomatitis virus (VSV), vesicular exanthema of swine virus (VESV), and swine vesicular disease virus (SVDV); therefore, these similar symptoms make SVA difficult to prevent and control.

The epidemic of SVA has seriously affected the development of the porcine breeding industry; therefore, research on the infection mechanism of SVA and the novel prevention and control methods of SVA will help to control the epidemic. SVA does not infect normal tissues of human, but it exhibits characteristic selective cytotoxic effects on neuroendocrine carcinoma, and does not lead to harmful non-target effects; therefore, SVA has been used for human oncolytic virus therapy after initial isolation, and extensive research on this virus is conducive to further treatment of SVA as an antitumor oncolytic virus to intervene in human cancer (13, 14).

Small non-coding RNA (sncRNA) includes transfer RNA (tRNA), PIWI-interacting RNA (piRNA), microRNA (miRNA), repeat-associated small interfering RNA (rasiRNA), small nucleolar RNA (snoRNA), and small nuclear RNA (snRNA) (15). Over the past two decades, significant advances have been made in the transcriptional and post-transcriptional regulatory mechanisms mediated by small RNAs. Based on studies of PIWI proteins, piRNAs were first identified in mouse testes in 2006. piRNAs are single-stranded small RNAs with 25 to 32 nucleotides. piRNAs are primarily expressed in germ cells and functionally depend on interacting with PIWI proteins and to form piRNA-induced silencing complexes (RISC). Current research on piRNA pathway mainly focus on transcriptional and post-transcriptional gene silencing. Evidence has suggested that the piRNA pathway induces gene silencing through heterochromatinization caused by DNA methylation or histone modification as well as cleavage of transposable elements (TEs) and protein-coding genes transcripts through RISC activity. In addition, piRNAs also provide diverse functions in tissue regeneration, tumor biology, and embryogenesis according to studies performed in somatic cells and cancer cells.

The piRNA is generally present in clusters on the chromosome. piRNA clusters can be transcribed to produce piRNA precursors. The location of piRNA clusters on the genome is conserved in related species. But interestingly the sequences of piRNAs differ even in closely related species, such as humans and chimpanzees, which indicates that the sequences of piRNAs have evolved very rapidly. Therefore, further studies on the transcriptome of piRNAs in different species are essential to better understand the regulatory mechanisms of piRNAs (16).

Previous studies have shown that type I interferons (IFNs) play an important role in the immune response during the early stage of SVA infection (17). Transcriptome analysis of SVA infected PK-15 cells suggested that RIG-I and IRF7 are the key factors in the induction of type III IFNs (18). However, there is no analysis of piRNA expression profile in SVA-infected hosts. In this study, we comprehensively analyzed the piRNA expression profiles of PK-15 cells either infected or not infected with SVA. A series of annotated and novel piRNAs from infected and non-infected samples were identified, and the potential regulatory effects of SVA on piRNA expression were explored.

## Materials and methods

### Virus and cells

Senecavirus A southwest strain was used to infect PK-15 cells (preserved in this laboratory). Cell culture in DMEM nutrient solution (Invitrogen, CA, USA) containing 10% FBS, which also contains 50 mg/ml penicillin/streptomycin antibiotic solution (Invitrogen, CA, USA), cultured under standard conditions of 5% CO_2_ and 37°C. PK-15 cells were infected by SVA, and cells were collected for high-throughput sequencing and stem ring PCR validation after infected for 24 h. The SVA VP1 gene forward primer: GCAGGTAACACTGACACCGA, reverse primer: GGCGGGGTGTTAGAAGACAA.

### RNA isolation

Total RNA was extracted from the above SVA-infected and non-infected PK-15 cells using Trizol reagent (Invitrogen, CA, USA). The purity and concentration of the total RNA sample were determined using a NanoPhotometer® spectrophotometer (IMPLEN, CA, USA). The integrity of total RNA samples was determined using the Agilent 2,100 Bioanalyzer system (Agilent Technologies, CA, USA).

### Small RNA library construction and sequencing

A total amount of 3 μg total RNA per sample was used as input material for the small RNA library. Sequencing libraries were generated using NEBNext® Multiplex Small RNA Library Prep Set for Illumina® (NEB, USA). Briefly, the piRNAs were ligated with 3′ and 5′ small RNA adapters and then converted to cDNA by reverse transcription using Illumina’s proprietary RT primers and amplification primers. PCR products were purified on an 8% polyacrylamide gel and bands corresponding to 140–160 bp were excised. Library quality was assessed on the Agilent Bioanalyzer 2,100 system using DNA High Sensitivity Chips according to the manufacturer’s instructions. The clustering of the index-coded samples was performed on a cBot Cluster Generation System using TruSeq SR Cluster Kit v3-cBot-HS (Illumina), following the manufacturer’s instructions. After cluster generation, the library preparations were sequenced on an Illumina HiSeq 2500/2000 platform. The 25–32 nt fragments obtained by high-throughput sequencing were used for piRNA analysis.

### Functional enrichment of piRNAs and data analysis

Differential expression analysis of two groups was performed using the DESeq R package (1.8.3). The *p*-values were adjusted using the Benjamini & Hochberg method. A corrected p-value of 0.05 was set as the threshold for significant DE by default. Gene ontology (GO) enrichment analysis was used on the target gene candidates of differentially expressed piRNAs. GOseq based Wallenius non-central hyper-geometric distribution, which could adjust for gene length bias, was implemented for GO enrichment analysis. KEGG is a database resource for understanding high-level functions and utilities of biological systems, such as the cell, organism, and ecosystem, from molecular-level information, especially from large-scale molecular datasets generated by genome sequencing and other high-throughput experimental technologies.^1^ We used KOBAS software to test the statistical enrichment of the target gene candidates in KEGG pathways.

### Stem-loop quantitative real-time PCR validation of differentially expressed piRNAs

Stem-loop qRT-PCR was used to confirm the significant DE piRNAs Uniq_38238, Uniq_38345, Uniq_38210, Uniq_38318, and Uniq_81367 in SVA-infected PK-15 cells. Significant DE piRNAs Uniq_85267, Uniq_88884, Uniq_78488, Uniq_121075, and Uniq_124874 in non-infected PK-15 cells were also confirmed by stem-loop qRT-PCR. Experiments were performed in triplicate on the ABI Prism 7900HT sequencing detection system (Applied Biosystems, Foster City, CA, USA). Data analyses were performed using a two-tailed Student’s *t* test. The specific forward primer of each piRNA and a universal reverse primer were used to quantitatively analyze the expression of piRNAs using U6 small nuclear RNA as a control.

### Statistical analysis

Quantitative real-time-PCR data are expressed as the mean ± standard deviation (SD) of at least three independent experiments. The paired Student’s *t* test was used to test the differences in sample means for data with normally distributed means. Statistical significance was assumed at *p* < 0.05.

## Results

### SVA infection in PK-15 cells

Quantitative real-time-PCR results showed that SVA VP1 mRNA expression level was the highest at 24 h after SVA infection. Therefore, PK-15 cells at 24 h after SVA infection were used for high-throughput sequencing (Supplementary Figure S1A). The SVA virus titer was 9.2 × 10^8^ TCID50/mL 24 h after infection. SVA infected PK-15 cells show an obvious cytopathic effect (CPE) and cell death (Supplementary Figure S1B).

### Overview of high-throughput sequencing data

**Figure 1:**
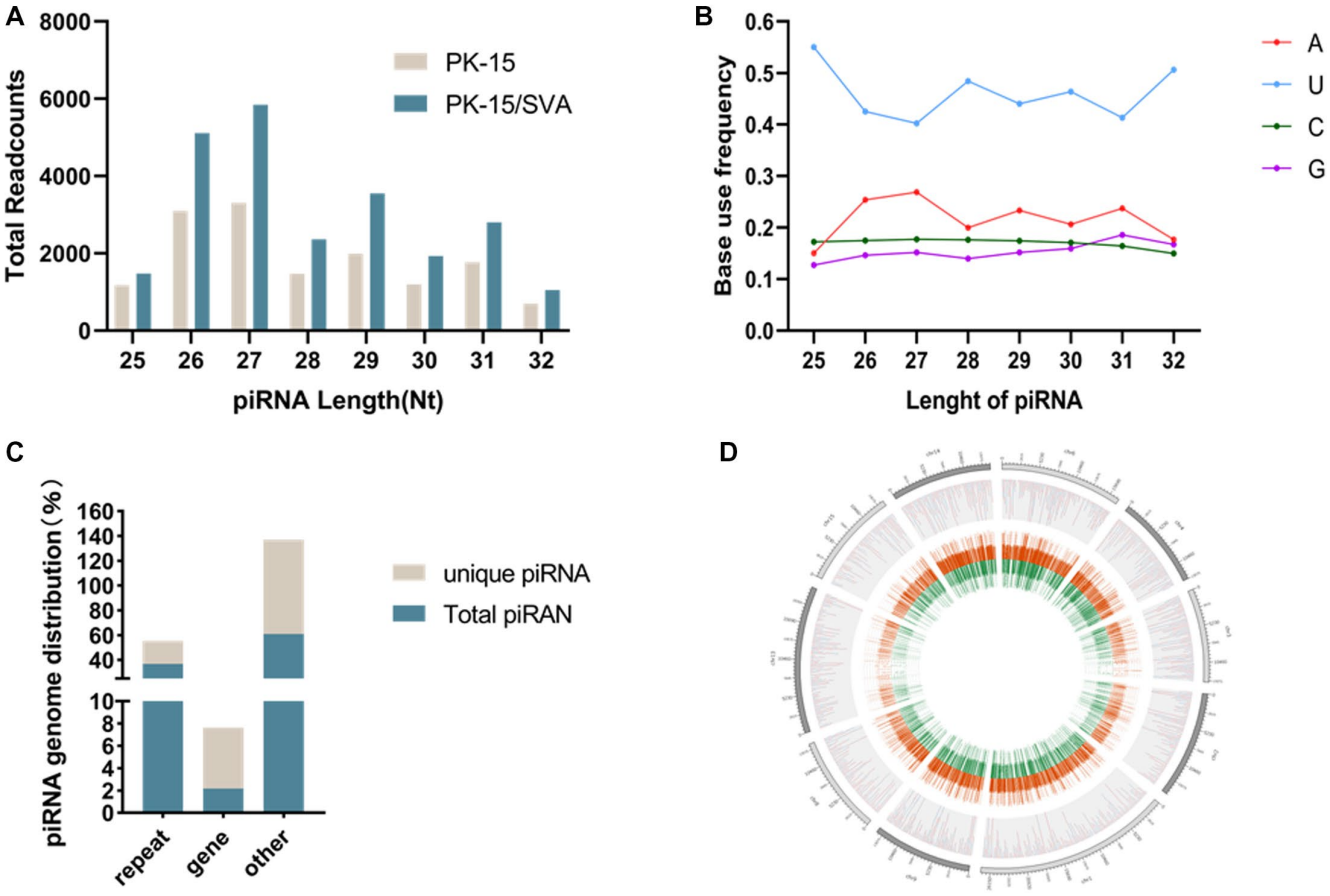
**(A)** Length distribution of PIWI-interacting RNAs (piRNAs). **(B)** First base preference of different length piRNAs. **(C)** Unique piRNA and piRNA source distribution. **(D)** piRNA chromosome density map.

To obtain the piRNA transcriptome of SVA-infected porcine cells, PK-15 cells were infected with SVA. SVA infection was confirmed by PCR, and piRNA expression profiles were generated using the Illumina HiSeq 2000 platform. Clean reads, adapter-trimmed reads, and non-infected PK-15 cells were generated (Supplementary Figure S2). The results demonstrated that the length of the piRNAs in SVA-infected and non-infected PK-15 cells ranged from 25 to 32 nucleotides (nt; Figure 1A). The 5′ terminal of piRNAs had a clear preference for U, and position 10 also had an intermittent preference for A (Figure 1B, Supplementary Figure S3). Interestingly, the piRNA expression level was significantly increased after SVA infection, and 24,120 piRNAs were identified in SVA-infected and non-infected PK-15 cells, of which 1,370 were novel piRNAs (Table 1). The relevant data has been uploaded to NCBI GEO (GSE222010).

**Table 1:** Overview of PIWI-interacting RNAs (piRNA) transcriptome data.

Samples	Raw reads	Clean reads	
		piRNA	piRNA cluster
PK-15	18,054	14,696	221
PK-15/SVA	28,614	24,120	316

*Raw reads is the original data and clean reads is the data after QC.

### The distribution and source of piRNAs

The mapping of piRNAs in the host genome was further examined to determine the source of piRNA biogenesis. The results demonstrated that 293,081 and 17,267 piRNAs (36.81 and 2.17% of the total piRNAs, respectively) mapped to the repeat and gene regions, respectively. Among the 72,385 unique piRNAs (9% of the total piRNAs), 13,388 and 3,945 unique piRNAs (18.50 and 5.45% of the unique piRNAs, respectively) mapped to the repeat and gene regions, respectively (Figure 1C，Supplementary Figure S4). However, the distribution of piRNAs in each chromosome was irregular but was not correlated with the length of chromosomes. Chromosomes 17, 5, 4, and 2 were piRNA-rich while chromosomes 1, 3, 16, 19, and X were piRNA deficient; the y chromosome contained almost no piRNA. Most piRNAs were concentrated in a small number of chromosomes (Figure 1D).

### Analysis of piRNAs with significant DE

**Figure 2:**
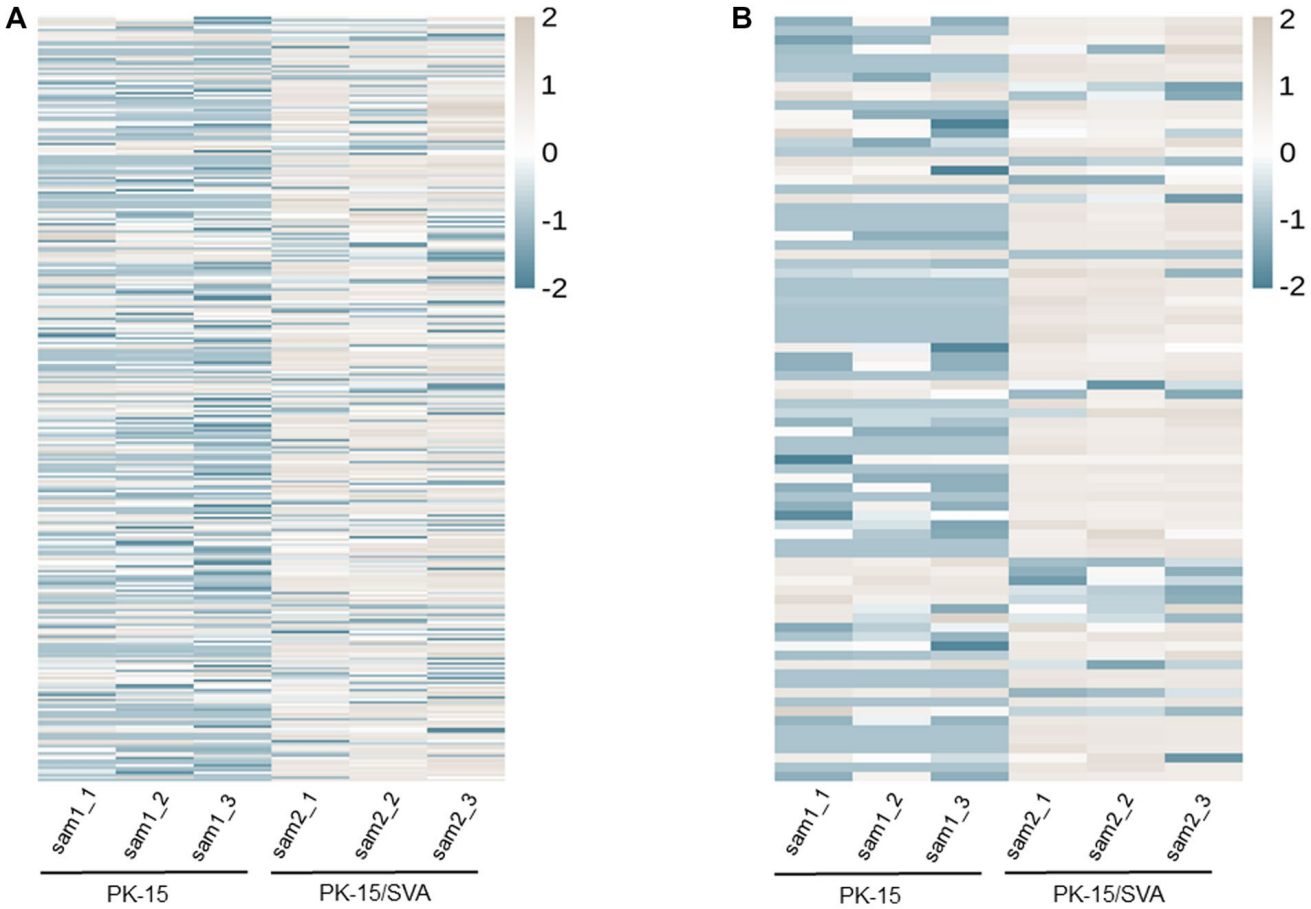
Heat map and hierarchical clustering of miRNA high-throughput sequencing data. Heat mapping and hierarchical clustering was used to analysis the PIWI-interacting RNA (piRNA) high-throughput sequencing data of non-infected and SVA-infected PK-15 cells based on their expression level. **(A)** piRNA heat map. **(B)** piRNA cluster heat map. The grey line indicates high relative expression, and the blue line indicates low relative expression.

**Figure 3:**
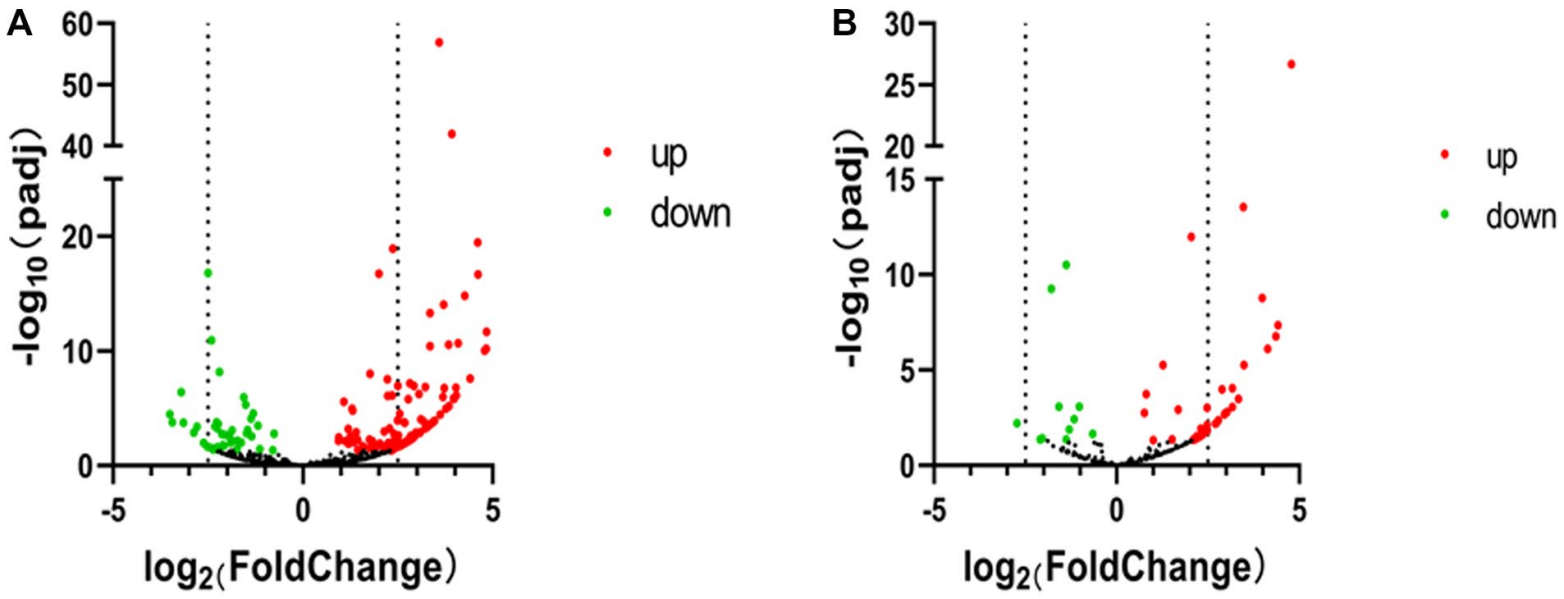
Volcano map of PIWI-interacting RNA (piRNA) high-throughput sequencing data. **(A)** Volcano map of piRNA expression between non-infected and SVA-infected PK-15 cells. **(B)** Volcano map showing variations in piRNA cluster expression between non-infected and SVA-infected PK-15 cells. The high-throughput sequencing data are graphed on a volcano map to visualize variations in piRNA expression. The values on the X and Y axes of the scatter plot are the normalized signal values for the samples (log2 scaled). The dashed lines are fold-change lines (the default fold-change value is 2.0). The expression of piRNA on the left side of the left dashed line or on the right side of the right dashed line was more than two-fold different.

**Figure 4:**
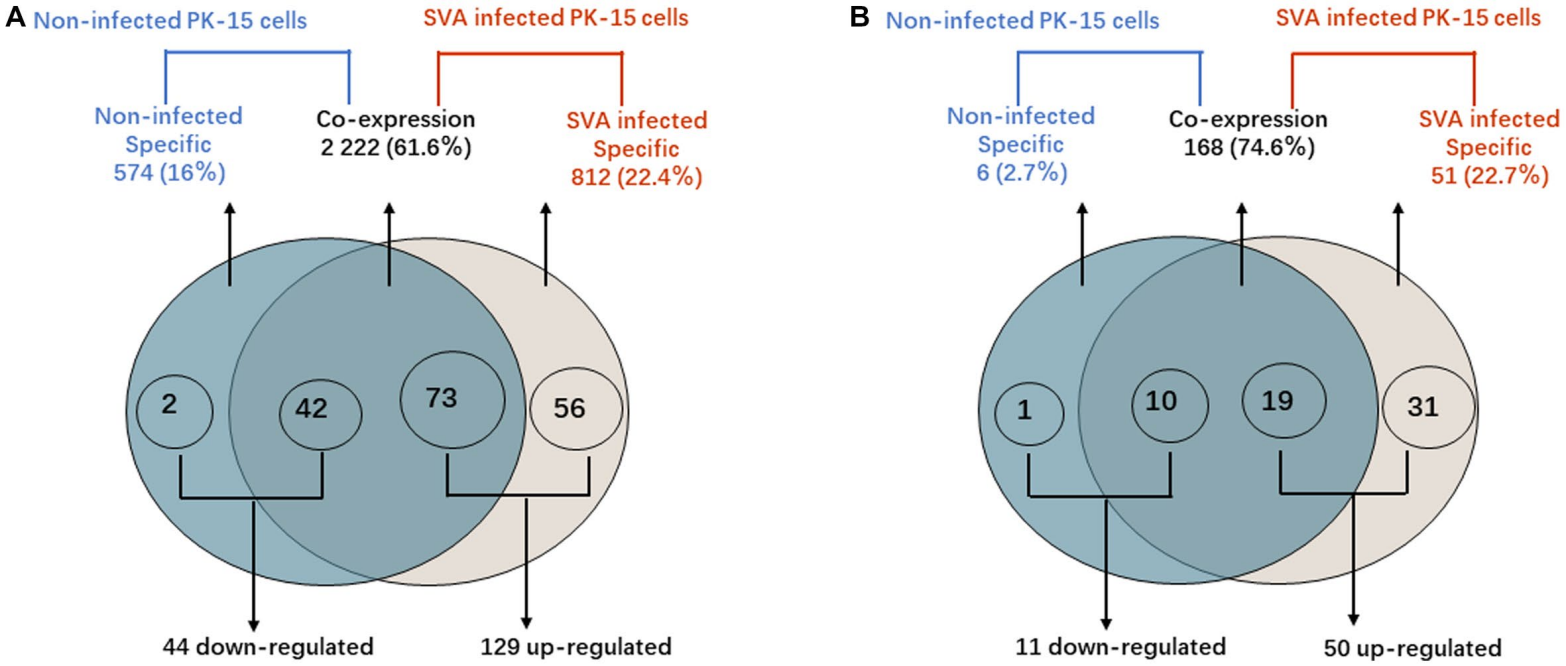
PIWI-interacting RNA (piRNA) differential expression analysis. **(A)** Venn diagram showing the distribution of 3,608 unique piRNAs between non-infected PK-15 cells and SVA-infected PK-15 cells. A total of 173 piRNAs with DE were identified in SVA-infected PK-15 cells. **(B)** Venn diagram showing the distribution of 225 piRNA clusters between non-infected and SVA-infected PK-15 cells. A total of 60 piRNA clusters with DE were identified in SVA-infected PK-15 cells.

According to the DE screening criteria (*q*-value <0.05 and | log_2_ (Foldchange) | > 1), the expression of piRNA and piRNA clusters in standardized inter-group samples were counted, and the piRNA and piRNA clusters with significantly changed DE were screened. To further study the effect of SVA infection on the expression of piRNAs, heat map (Figure 2A) and volcano map (Figure 3A) analysis were performed. Corresponding analysis was also conducted on the piRNA clusters (Figures 2B, 3B). 812 (22.4%) and 574 (16%) piRNAs were found to be specifically expressed in SVA-infected and non-infected PK-15 cells, respectively (Figure 4A). A total of 225 piRNA clusters were identified, of which 51 (22.7%) and 6 (2.7%) were specifically expressed in SVA-infected and non-infected PK-15 cells, respectively (Figure 4B). Compared with DE in the non-infected group, there was significant DE of 173 piRNAs after SVA infection, of which 129 were significantly upregulated and 44 were significantly downregulated (Figure 4A). The number of piRNA clusters with significant DE was 61, of which 50 were significantly upregulated and 11 were significantly downregulated (Figure 4B).

These findings indicated that the expression level of piRNAs in PK-15 cells was significantly changed after infection with SVA, indicating that SVA may enhance piRNA transcriptional activity.

### qRT-PCR confirmation of differentially expressed piRNAs

**Figure 5:**
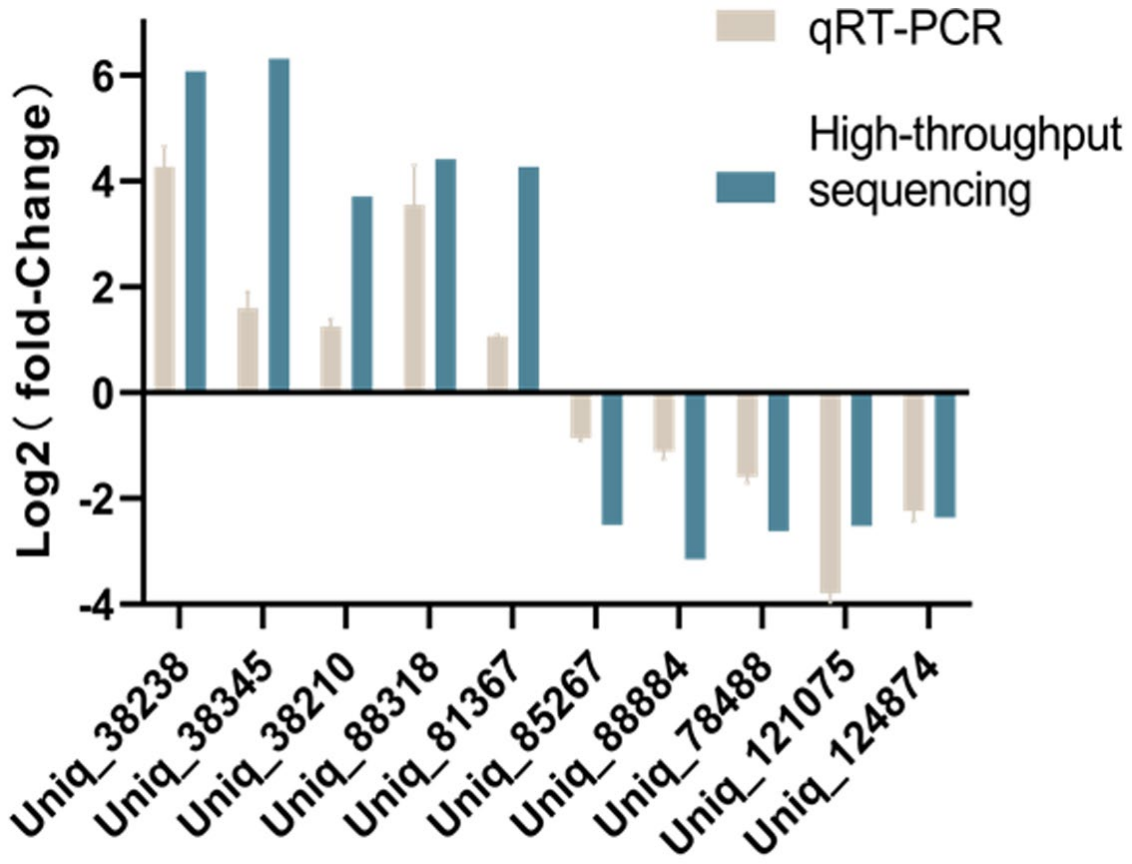
Comparison of qRT-PCR and high-throughput sequencing results.

Stem-loop qRT-PCR was used to validate the expression levels of piRNAs with DE in non-infected or SVA-infected PK-15 cells. The expression levels of novel SVA-encoded miRNAs were also confirmed. Stem-loop qRT-PCR results were generally consistent with those of high-throughput sequencing (Figure 5).

### Functional annotation of piRNA-generation genes

Previous studies suggested that piRNAs must rely on RISC formed with PIWI.

protein to perform their function of cleaving TEs and protein-coding gene transcripts. GO annotation on piRNA-generating genes was used to explore the potential regulatory roles of the piRNAs during SVA infection. The GO results proved that the piRNA-generation genes specific to SVA-infected PK-15 cells were significantly related to biological regulation of intracellular, RNA binding, and cellular component in metabolic processes.

**Figure 6:**
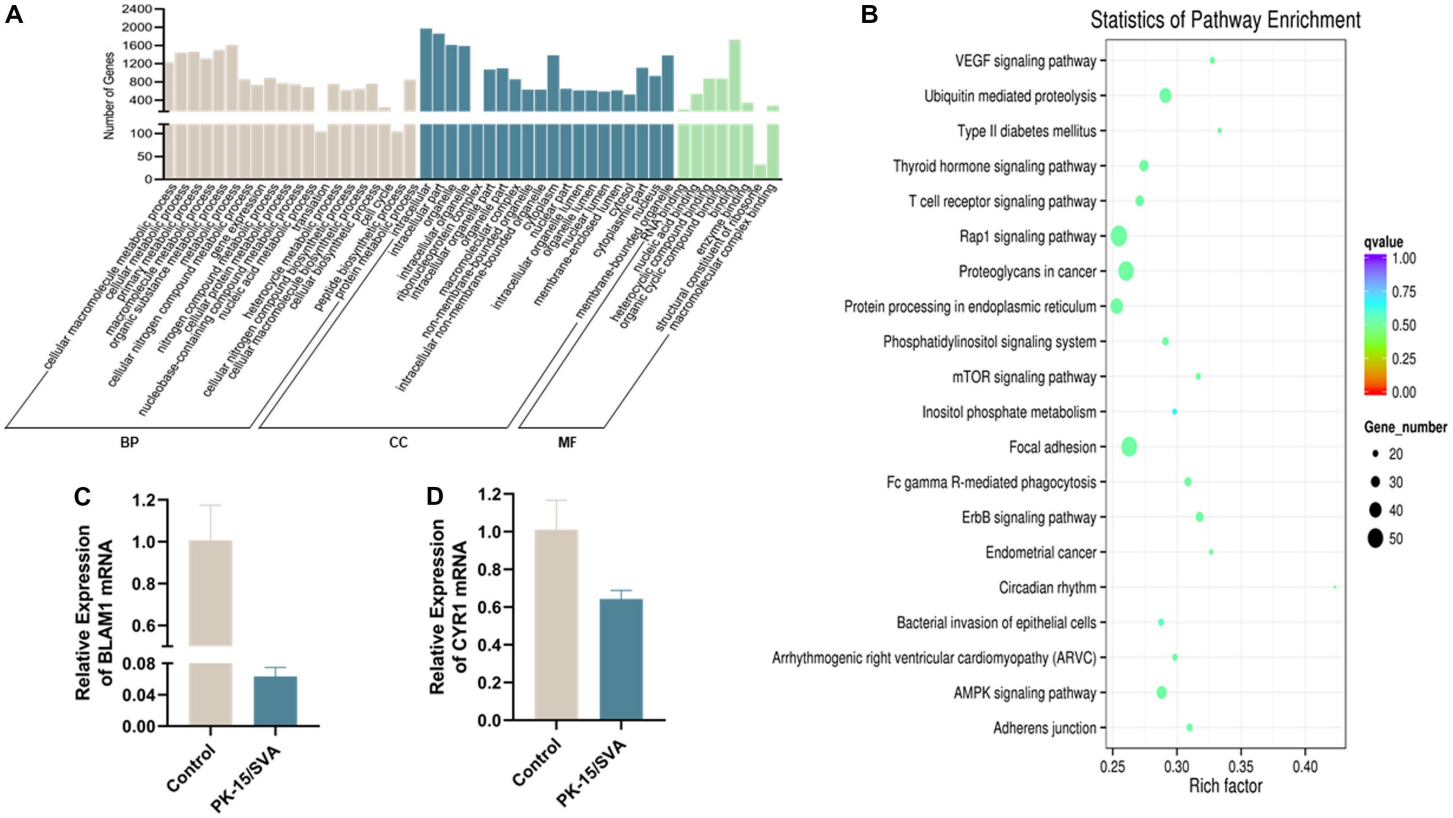
Gene ontology (GO) annotation of piRNA target genes. **(A)** GO annotation of differentially expressed piRNA target genes in SVA-infected PK-15 cells (vs. non-infected PK-15 cells). **(B)** Scatter plot of candidate target gene KEGG enrichment. The vertical axis represents the pathway name, horizontal axis represents the rich factor, size of the dot indicates the number of candidate target genes in this pathway, and color of the dot corresponds to different *Q*-value ranges. **(C)** Transcriptional level of BMAL1 was detected by qRT-PCR. **(D)** Transcriptional level of CRY1 was detected by qRT-PCR.

In organisms, different genes coordinate with each other to exercise their biological functions, and pathway significance enrichment can determine the most important biochemical metabolic pathways and signal transduction pathways in which candidate genes are involved. We plotted the sequencing data into a candidate target gene KEGG enrichment scatter point map. The results revealed that the top three pathways with the highest degree of enrichment were circadian rhythm, type II diabetes mellitus, and VEGF signaling pathway. In this study, we found that the expression level of main piRNA-generation genes *BMAL1* and *CRY1* were significantly downregulated after SVA infection, which indicated that SVA infection did have an effect on circadian rhythm in PK-15 (Figure 6).

## Discussion

The clinical symptoms of SVA infection are similar to those of FMDV, VSV, VESV, and SVDV, although there is no effective commercial prevention and control technology for SVA. The diarrhea of piglets under 7 days of age and the increase of neonatal mortality due to SVA infection are reported, and the expanding epidemic has caused huge losses to the breeding industry (2, 19). Although progress has been made in studying the immune escape mechanism of SVA in host cells, the interaction between SVA and host at the epigenetic level has not been fully clarified. Further research is needed to fully understand the complex molecular mechanisms of the innate immune response to SVA.

PIWI-interacting RNA is highly conserved among species, which is mainly reflected in length distribution and base bias (20). piRNA length distribution is typically between 21 and 31 nt; for example, the length of piRNA identified in human gastric cancer cells is between 25 and 31 nt while the length of piRNA identified in mice ranged from 26 to 31 nt (21). Similar to recent studies, the length of piRNA in SVA-infected PK-15 cells was between 25 and 32 nt, with the largest transcription level of piRNAs in the length range of 26–31 nt. This resembles the length distribution of the above species’ piRNAs, indicating that the length distribution of piRNAs of different species is highly conserved. In organisms that have the ability to generate piRNA, it has been observed that piRNA is generated through conventional unidirectional transcription from single-stranded clusters. The sequence characteristics of piRNA exhibit a notable degree of stability across various species. Additionally, it has been observed that the first base at the 5′-end of piRNA shows a bias toward uracil (U), a trait that has been verified in different animals like *mice*, *Silkworms*, and *Drosophila* (21, 22). The first base of piRNA of the 25–32 nt region in this study was mainly biased toward U, which provides another example of the first base bias of piRNA.

The regulation of gene expression by piRNA is mainly reflected at two levels, transcriptional and post-transcriptional regulation. Transcriptional regulation regulates gene expression primarily through epigenetic modifications, turning on or off gene expression through modifications to chromosomes and DNA. Modifications in chromosomes necessarily lead to changes in gene expression, and long non-coding RNA (lncRNA) is one type of RNA that is transcribed from between genes. Approximately 66% of lncRNAs are known to contain reversed possum sequences, so it is speculated that lncRNAs are most likely regulated by piRNAs (23). A study of rat Rasgrf1 sequences found that the methylation process of Rasgrf1 sequences requires the participation of piRNAs. In lncRNA, transposon sequences from Rasgrf1 are recognized by the piRNA, enabling regulation of the lncRNA (24).

Post-transcriptional regulation is mediated through the complementary recognition of piRNAs and transcripts. This enables the degradation or cleavage of mRNA to reduce the number of transcript RNAs, thereby achieving regulation of their expression. Studies in mice by Gou et al. (25) have shown that CAF1 can bind to a piRNA/MIWI complex that can lead to deadenylation of target mRNAs recognized by piRNA, eventually causing mRNA degradation. Zhang et al. (26) reported on the pRNA/MIWI-mediated shearing mechanism of mRNA.

Recent studies have shown that SVA infection may affect host immune and inflammatory responses, but the role of piRNA in SVA infection and immunosuppression is not clear (27). Therefore, to explore the potential regulatory role of piRNAs in SVA infection, we constructed SVA-infected PK-15 cells model and analyzed the expression profile of host piRNAs after SVA infection via RNA-Seq and bioinformatic methods. Under the stress of SVA infection, DE of 129 piRNAs in PK-15 cells was significantly upregulated while DE of 44 piRNAs was significantly downregulated compared with DE in the non-infected group; 56 of the specifically upregulated piRNAs were expressed only in SVA-infected PK-15 cells.

This suggests that DE piRNA may be widely involved in SVA infection and antiviral immunity. In addition, recent studies have shown that early SVA infection affect host proteome and host cell signaling pathways (28). Therefore, it is necessary to further study the interaction between significant DE piRNAs and host signaling pathway. Through GO enrichment and KEGG pathway analysis of differentially expressed genes after SVA infection, the PK-15-specific piRNA-producing hormone gene of SVA infection was mainly concentrated in the Biological process and cellular component, whereas the enrichment degree of molecular function was relatively low, indicating that after SVA infection of PK-15 cells, the host cell becomes more active in metabolism, proliferation, and differentiation and that changes have occurred in the cell.

KEGG signaling pathway analysis indicated that the piRNAs’ source genes were enriched in the focal adhesion pathway after SVA infection. Focal adhesion mediates the attachment of cells to the extracellular matrix and is associated with the fixation of cells to the extracellular matrix. Focal adhesion facilitates maintenance of cellular tension and cell signaling during motility. Focal adhesion kinase (FAK), a focal adhesion tyrosine kinase that mediates signaling between the extracellular matrix and the cytosol, serves as an antiviral component of the RIG-1-like pathway by orchestrating the activation of mitochondrial antiviral signaling (MAVS) (29). FAK phosphorylation was important for transport of herpes simplex virus capsids to the nuclear pore (30). SVA infection leads to changes in the cytoskeleton of actin, and cellular perception of these cytoskeletal changes may lead to modulation of the expression of piRNAs.

The ubiquitin-mediated pathway is a specific protein degradation pathway that is tightly regulated in space and time. E2 ubiquitin-conjugating enzyme UBE2L6 promotes Senecavirus A proliferation by stabilizing the viral RNA polymerase (31). The production of type I IFNs are hindered by SARS-CoV-2 membrane protein through the degradation of TBK1 via ubiquitin-mediated processes (32). M1 of influenza A virus (IAV) is important for the virus life cycle, PSMD12 interacts with M1 and mediates K102-linked ubiquitination of M1 at the K63 locus, thereby positively regulating influenza virus proliferation (33). It can be inferred that viral infection has the capability to circumvent intrinsic immunity by stimulating self-replication through host ubiquitin-mediated proteolysis pathway. Significant DE piRNAs’ source genes were enriched in the ubiquitin-mediated proteolysis pathway after infection with SVA. In light of this, piNRAs may have a regulatory effect on SVA replication.

KEGG analysis disclosed that significant DE piRNAs source genes are mainly enriched in the pathway of circadian rhythms. The abnormal change or disorder of circadian rhythm is closely related to the occurrence and development of many cancers (34, 35). The imbalance of cell proliferation and apoptosis is a fundamental cause of cancer (36). Studies have shown that the circadian clock gene *BMAL1* affects dendritic cells (DCs) and CD8 T cells through the co-stimulatory molecule CD80, thereby exerting circadian anti-tumor functions that control melanoma volume (37). Cryptochrome 1 (CRY1) is an evolutionary conserved transcriptional coregulator, identified as a tumor-specific regulator of DNA repair (38). *CRY1* is also a core circadian gene, modulates the circadian rhythm and promotea the development of cancer (39). SVA has become a popular oncolytic virus in recent years, and the relationship with cancer is also closely related. SVA has been shown to inhibit the proliferation of small cell lung cancer. (36). Therefore, we selected *BMAL1* and *CRY1* for qRT-PCR validation and found that the expression of *BMAL1* and *CRY1* was significantly downregulated after SVA infection, indicating that after viral infection, piRNAs may exert an effect on circadian rhythm. Changes in circadian rhythm will lead to abnormalities in cells and, consequently, abnormal proliferation or death of cells, thus becoming a cause of anti-cancer. Therefore, future studies should focus on selecting piRNAs with significantly changed DE for the synthesis of mimics and inhibitors and verifying the functional changes in piRNAs induced by overexpression and knock out in multiple porcine cell lines. Thus, further study on the regulatory mechanism of DE piRNA on the apoptosis and cancer process after SVA infection is conducive to the application of SVA. An in-depth study of circadian rhythm may provide new ideas and methods for cancer treatment, which may also become the theoretical basis for SVA as an oncolytic virus.

## Conclusion

To summarize, we first obtained piRNA expression profiles in SVA-infected PK-15 cells based on RNA-seq. Bioinformatic analysis showed that SVA infection altered the expression profile of piRNAs, and 173 significantly DE piRNAs were found. Functional annotation analysis revealed that the source genes of significantly DE piRNAs are enriched in signaling pathways related to host immunity, intracellular homeostasis and tumor activities responses. In addition, we found that the expression levels of the major piRNA-generating genes *BMAL1* and *CRY1* were significantly downregulated after SVA infection. This suggests that SVA may affect circadian rhythm and promote apoptosis by inhibiting the major piRNA-generating genes *BMAL1* and *CRY1*. These findings have implications for understanding the anti-tumor mechanism of SVA and for the prevention and control of SVA in livestock breeding.

## Data availability statement

The datasets presented in this study can be found in online repositories. The name of the repository and accession number can be found at: NCBI; GSE222010.

## Author contributions

XL and CW designed and directed the project. CW, YC, XWY, YD and XL, performed the experiments and analyzed the data. XL, WL, YY, ZX, YZ, XY, XW, CZ and SL, provided reagents and helped data analysis, experimental interpretation, and drafting revision. CW wrote the manuscript with comments from all authors. All authors contributed to the article and approved the submitted version.

## Funding

This study was supported by Chongqing Basic Research and Frontier Technology Project (cstc2020jcyj-msxm1458) (XL) and (cstc2021jscx-dxwtBX0007) (ZX), Sichuan Province Regional Innovation Cooperation Project (2022YFQ0023) (XL), Sichuan Provincial “Fourteenth Five Year Plan” (2021ZDZX0010) (ZX and XL), National Science Foundation of China (No. 82260125), Science Foundation of Hainan Province (No. 822MS181), Key project of Anhui Province (No. S202104j07020097) and the project supported by Hainan Province Clinical Medical Center. Open subject of State Key Laboratory of Silkworm Genome Biology (SKLSGB-ORP202104).

## Conflict of interest

YZ, XY, XW, CZ, and SL were employed by Veterinary Biologicals Engineering and Technology Research Center of Sichuan Province, Animtech Bioengineering CO., LTD.

The remaining authors declare that the research was conducted in the absence of any commercial or financial relationships that could be construed as a potential conflict of interest.

## Publisher’s note

All claims expressed in this article are solely those of the authors and do not necessarily represent those of their affiliated organizations, or those of the publisher, the editors and the reviewers. Any product that may be evaluated in this article, or claim that may be made by its manufacturer, is not guaranteed or endorsed by the publisher.

## Footnotes

^1^ http://www.genome.jp/kegg/
