# Supplementary material for: The porcine piRNA transcriptome response to Senecavirus a infection
Source: Front Vet Sci. 2023 May 30;10:1126277. doi: 10.3389/fvets.2023.1126277 (PMC10265626; doi:10.3389/fvets.2023.1126277)
Supplement: Supplementary file 1 [file Data_Sheet_1.pdf]

Supplementary Figure Legend

Supplementary Figure S1. The PK-15 cells were infected with the SVA

A. mRNA expression level of SVA VP1 at 0-72h after SVA infection in PK-15 cells.

B. PK-15 cells and PK-15 cells infected with SVA at 24h.

A.

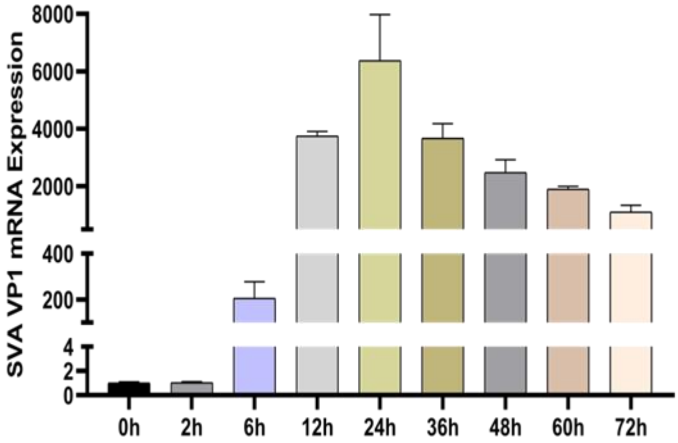

B.

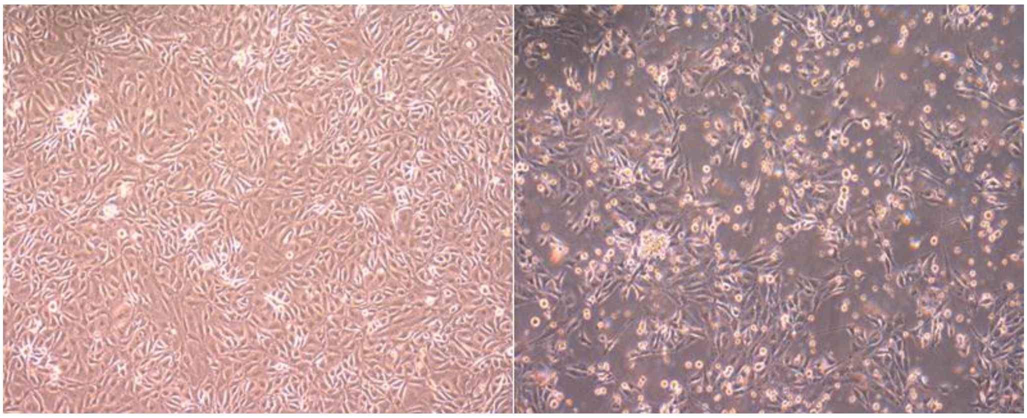

PK-15 cells

PK-15 cells infected with SVA at 24h

Supplementary Figure S2. Schematic diagram of analysis flow of piRNA.

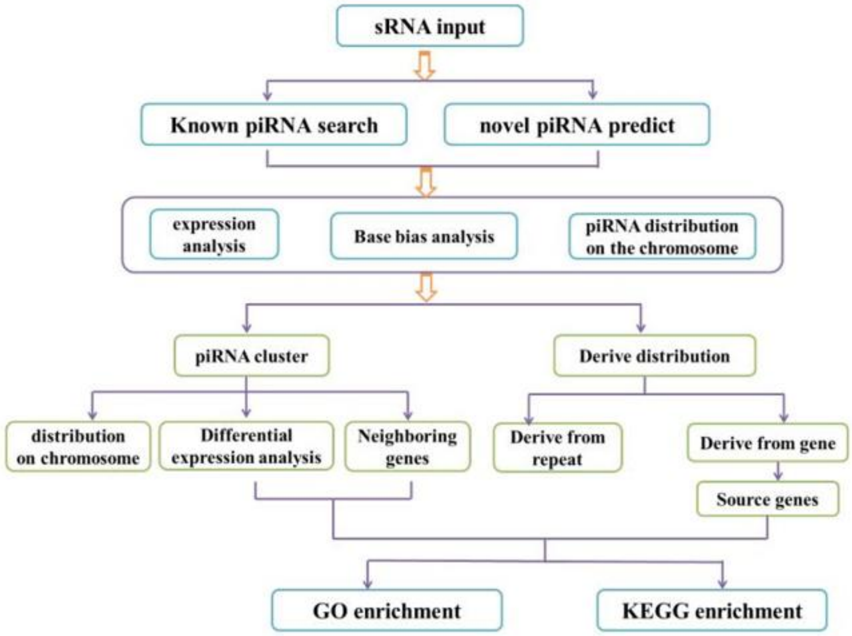

Supplementary Figure S3. Schematic diagram of base preference of each site of piRNA sequence.

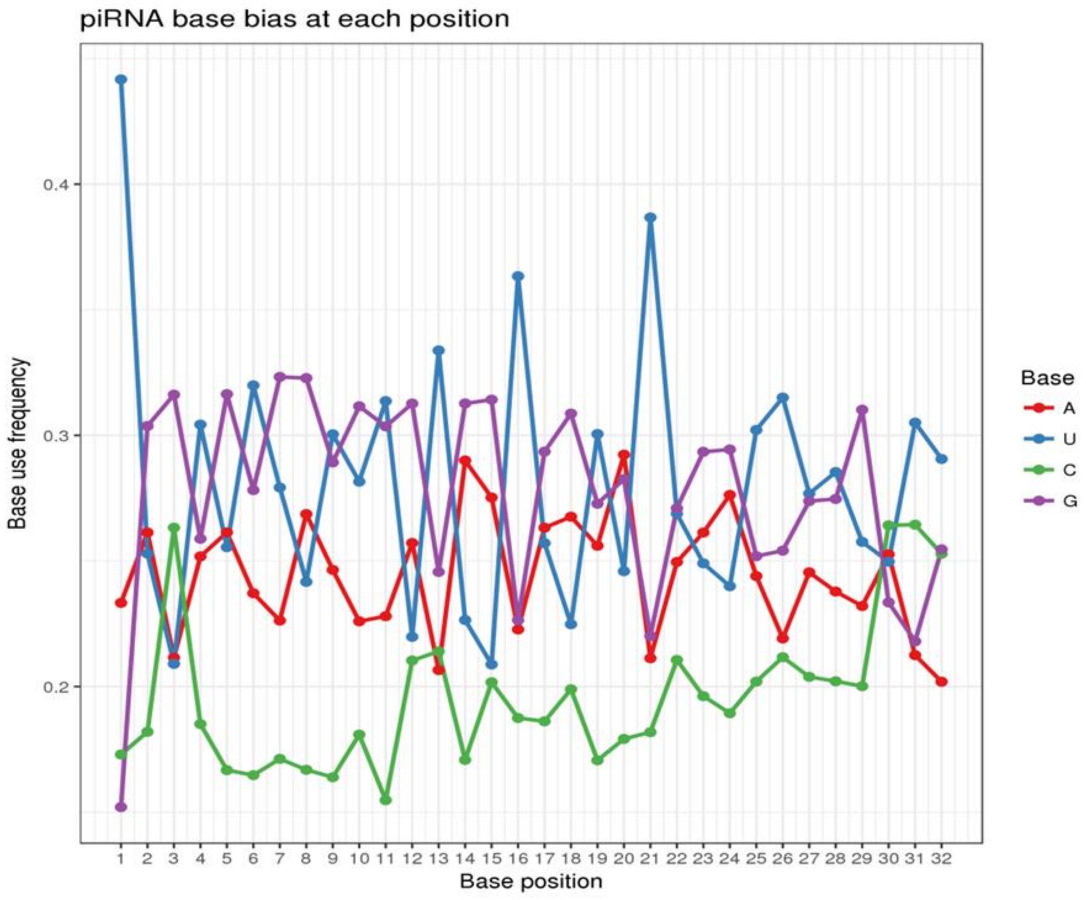

Supplementary Figure S4. (A) Schematic Diagram of piRNA Source Distribution. (B) Schematic Diagram of unique piRNA Source Distribution.

A.

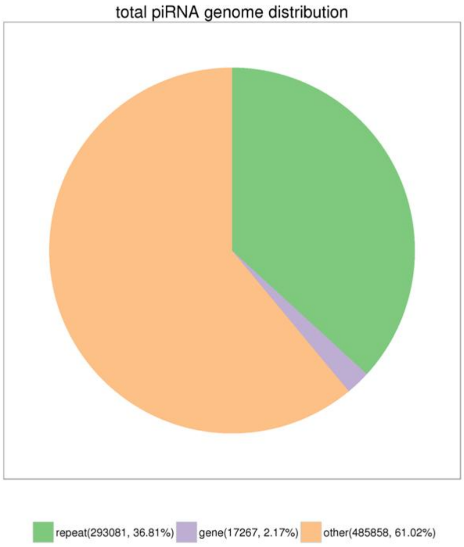

B.

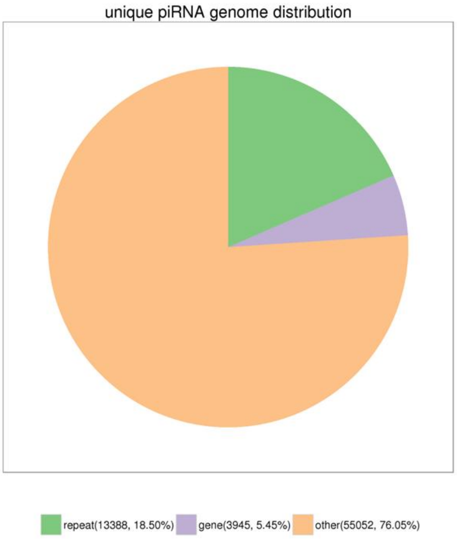

Supplementary Table S1. Primers of 10 DE piRNAs in PK-15 cells

Characteristics of 10 DE piRNAs in PK-15 cells

piRNA id	Sequence	Primer-F	Primer-R
Uniq_38238	AACTGACTGGCACCATAGAAGAATTGATT	GCAACTGACTGGCACCATAGAA	AACGCTTCACGAATTTGCGT
Uniq_38345	AACTGACTGGCACCATAGAAGAATTG	GCAACTGACTGGCACCATAGAA	
Uniq_38210	AACTGACTGGCACCATAGAAGAATTGA	GCAACTGACTGGCACCATAGAA	
Uniq_38318	AACTGACTGGCACCATAGAAGAATTGATT	GCAACTGACTGGCACCATAGAA	
Uniq_81367	AACTGTGGTAATTCTAGAGCTAATAC	GCGAACTGTGGTAATTCTAGAGCT	
Uniq_85267	TGTAAACATCCTTGACTGGAAGCTTT	GCTGTAAACATCCTTGACTGGAAG	
Uniq_88884	AGCAGCATTGTACAGGGCTATGAAC	AGCAGCATTGTACAGGGCTATG	
Uniq_78488	CTTCGGTGATTAGTCTCAACTGACTGACA	GCCTTCGGTGATTAGTCTCAACT	
Uniq_121075	GATGATGATCAAAGATCTGATATTCTGTTT	GCGGATGATGATCAAAGATCTG	
Uniq_124874	CAGGTCTGTGATGCCCTTAGATGTTC	GCAGGTCTGTGATGCCCTTAG	
